# Myocardial oxidative stress correlates with left ventricular dysfunction on strain echocardiography in a rodent model of sepsis

**DOI:** 10.1186/s40635-017-0134-5

**Published:** 2017-04-12

**Authors:** Bereketeab Haileselassie, Erik Su, Iraklis Pozios, Diego F. Niño, Hongyun Liu, Dai-Yin Lu, Ioannis Ventoulis, William B. Fulton, Chhinder P. Sodhi, David Hackam, Brian O’Rourke, Theodore Abraham

**Affiliations:** 1 Department of Anesthesiology and Critical Care Medicine, The Johns Hopkins University School of Medicine, Baltimore, MD USA; 2 Department of Medicine, Johns Hopkins University School of Medicine, Baltimore, MD USA; 3 Department of Surgery, Johns Hopkins University School of Medicine, Baltimore, MD USA; 4 Department of Pediatrics, Division of Critical Care, Stanford University School of Medicine, 770 Welch Road, Suite 435, Palo Alto, CA 94304-5731 USA

**Keywords:** Sepsis-induced myocardial dysfunction, Shock, Ultrasound, Sepsis

## Abstract

**Background:**

Recognition of cardiomyopathy in sepsis can be challenging due to the limitations of conventional measures such as ejection fraction (EF) and fractional shortening (FS) in the context of variable preload and afterload conditions. This study correlates myocardial function using strain echocardiography (SE) with cardiomyocyte oxidative stress in a murine model of sepsis.

**Methods:**

C57BL/6J mice were randomized into control (*n* = 10), sham (*n* = 25), and a cecal ligation and puncture (CLP) (*n* = 33) model of sepsis. Echocardiography was performed pre-, 12, 24, and 48 h post-injury. Cardiac pro-inflammatory cytokines and mitochondrial redox scavenger expression were evaluated in a subset of each arm. To evaluate the influence of redox scavenger upregulation on oxidative injury and cardiac function, CLP was performed on mitochondrial catalase-upregulated C57BL/6J MCAT^+/+^ mice (*n* = 12) and wild-type (WT) animals for comparison.

**Results:**

Septic C57BL/6J mice exhibited depressed longitudinal strain (LS) when compared to sham and control at 24 h (*p* < 0.01) and 48 h (*p* = 0.04) post-CLP despite having a preserved EF. Furthermore, there was a significant association between increased odds of mortality and depressed LS (OR = 1.23, *p* = 0.04). Septic C57BL/6J mice concomitantly demonstrated increased expression of cardiomyocyte pro-inflammatory cytokines and decreased expression of redox scavengers at 24 and 48 h.

When comparing C57Bl/6 MCAT^*+/+*^ mice and C57BL/6J WT mice, a significant decrease in LS was identified in the WT mice at 24 h (MCAT = −23 ± 5% vs. WT = −15 ± 4% *p* < 0.01) and 48 h (MCAT = −23 ± 7% vs. WT = −15 ± 4.3% *p* = 0.04) post-CLP which correlated with significant increase in the level of cardiac oxidative stress following CLP.

**Conclusions:**

In this sepsis model, SE identified cardiomyopathy despite normal EF. SE depression temporally coincides with upregulation of inflammatory cytokines and decreases expression of key mitochondrial ROS scavengers. Upregulation of redox scavenger (CAT) abrogates oxidative stress and cardiac dysfunction in this sepsis model.

**Electronic supplementary material:**

The online version of this article (doi:10.1186/s40635-017-0134-5) contains supplementary material, which is available to authorized users.

## Background

Despite major advances in management of the critically ill, sepsis continues to be a major cause of morbidity and mortality worldwide [1]. This is demonstrated by the increasing prevalence of sepsis over the last two decades [1, 2]. In the progression of septic shock, the role of sepsis-induced myocardial dysfunction (SIMD) is becoming increasingly apparent [3, 4], with data suggesting an association between mortality and cardiac dysfunction in sepsis [5, 6].

Over the last 40 years, diagnostic criteria for SIMD have evolved. Currently, two-dimensional (2D) echocardiography is the most common diagnostic tool for assessing myocardial function in sepsis. This is mainly performed with dimension-based quantitative metrics such as fractional shortening (FS) and ejection fraction (EF) [7]. Although this measure of left ventricular systolic function is frequently used, recent observational studies have underscored the presence of biventricular systolic and diastolic dysfunction in septic patients [8–10].

Strain echocardiography (SE) is a modality for evaluating myocardial function that is thought to be less dependent on preload and afterload [11]. Unlike dimension-based metrics (EF and FS), SE evaluates cardiac function by tracking cardiac tissue deformation throughout the cardiac cycle [11]. Although SE has been successfully used to identify myocardial compromise in various cardiovascular diseases [12–14], limited data exists regarding the utility of SE in the context of sepsis. Accordingly, this study investigates the development of SIMD using SE in a well-established murine model of sepsis [15] and evaluates the association between cardiomyopathy identified by SE and key mechanistic pathways involved in SIMD.

## Methods

### Animal surgeries

This investigation was approved by the Johns Hopkins University Animal Care and Use Committee (protocol number ra14m360). After a 5-day acclimation period, 14-week male C57BL/6J mice (Charles River Laboratory, Wilmington, MA) were randomized into control (*n* = 10), sham (*n* = 25), and CLP model of animal sepsis (*n* = 33). The CLP procedure was performed according to the method described by Rittirsch and colleagues [15]. All mice were anesthetized with isoflurane during the procedure (induction: 3–5% and maintenance: 1–2 ). A 1-cm midline laparotomy incision was made to expose the cecum and adjoin intestine. The cecum was then ligated tightly 5 mm from its base and subsequently punctured once with a sterile 18-gauge needle on the anti-mesenteric border. The bowel was then returned to the peritoneal cavity, and the abdominal incision was closed with a running 4.0 silk suture.

Sham-operated mice underwent laparotomy as described above without CLP. Volume resuscitation in the form of 60 mL/kg of 0.9% normal saline was administered to all mice.

### Echocardiography

**Fig. 1:**
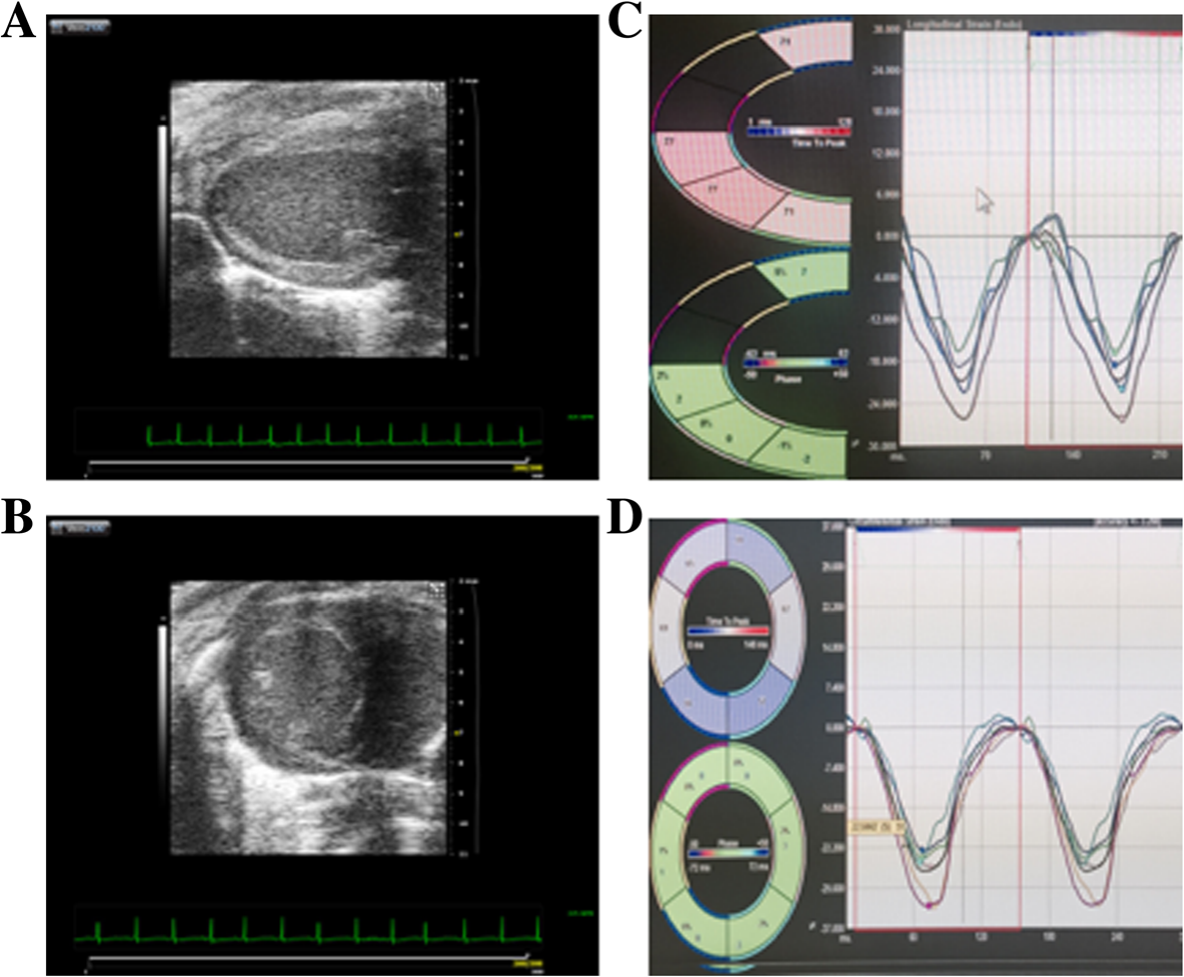
Strain analysis by echocardiogram post-processing software (VevoStrain, FUJIFILM VisualSonics Inc.). The parasternal long access view (**a**) was used to calculate the longitudinal strain (LS, %) (**b**) and the longitudinal strain rate (LSR, 1/sec). The parasternal short axis view (**c**) was used to calculate the circumferential strain (CS, %) (**d**) and circumferential strain rate (CSR, 1/sec)

Images were obtained with a Vevo 2100 ultrasound machine equipped with linear array transducer (FUJIFILM VisualSonics Inc., Toronto, Canada). Echocardiography was performed pre- as well as at 12, 24, and 48 h post-surgery to assess cardiac function. SE analysis was performed with speckle-tracking strain post-processing software (VevoStrain, FUJIFILM VisualSonics Inc., Toronto, Canada). The parasternal long axis view of the heart was used to measure longitudinal strain (LS), longitudinal strain rate (LSR) as well as EF via Simpson’s method (Fig. 1). The parasternal short axis view was used to measure circumferential strain (CS) and circumferential strain rate (CSR) (Fig. 1) [11].

The apical view was utilized to attain tissue and pulse wave Doppler across the mitral annulus. The ratio of peak flow velocity across the mitral annulus during early and late diastole (E/A) and the ratio between mitral inflow and mitral annular excursion velocity during early diastole (E/e’) were used as metrics of diastolic dysfunction. Echocardiography was attained by one blinded physician (BH) utilizing standard sedation and image acquisition protocol while two blinded physicians (BH and HL) analyzed the echocardiographic studies.

### Quantitative real-time PCR

A subset of C57BL/6J mice from each group were euthanized at 24 h (*n* = 15) and 48 h (*n* = 16) post-CLP to evaluate cardiac pro-inflammatory cytokine expression profiles. Total RNA was isolated from a transverse section of myocardial tissue and reverse transcribed using the QuantiTect Reverse Transcription Kit (Qiagen). Gene-specific cDNA was amplified and quantified in a real-time thermal cycler system using specific primer pairs (Additional file 1: Table S1). Water was used as a non-template control. The results were normalized using the housekeeping gene *Rplp0*.

### DHE staining

Cellular oxidative stress was evaluated on the left ventricular tissue using Dihydroethidium (DHE) staining (cell-permeable fluorescent redox indicator). Transverse sections of myocardial tissue were placed in Tissue-Tek optimal cutting temperature compound (Miles Laboratories; Elkhart, IN), and frozen at −80 °C. 5−10 μM cryo-sections were prepared from the frozen myocardial tissue and were irrigated using PBS. DAPI (4′,6-diamidino-2-phenylindole), a cell-permeable fluorescent nuclear stain was applied to the tissue and incubated for 10 min. DHE staining was then applied to each tissue section and incubated in a light-protected incubator at 37 °C for 30 min. DHE uptake was evaluated using confocal florescence microscopy (Nikon Instruments Inc. Tokyo, Japan) and was quantified using ImageJ processing software (FUJIFILM Inc., Minato, Tokyo, Japan).

### Mitochondrial catalase upregulated (MCAT^+/+^) transgenic mice

To evaluate the influence of free-radical scavenger mitochondrial catalase (CAT) overexpression on cardiomyocyte ROS dynamics and cardiac function, MCAT knock-in (C57Bl/6 MCAT^*+/+*^) transgenic mice (*n* = 12) (Jackson Laboratories; Bar Harbor, ME) were subjected to CLP. MCAT knock-in mice were compared to a subset of C57BL/6J wild-type (WT) mice (*n* = 14) which simultaneously underwent CLP model. SE was measured pre-, 24 h, and 48 h post-CLP and oxidative stress was assessed by DHE staining.

### Statistical analysis

Data distribution was evaluated with kernel density plots and the Shapiro-Wilk test for normality. Differences between groups over time were compared by one-way analysis of variance (ANOVA) with repeated measures for normally distributed variables and Friedman test for non-parametric variables. Variables that had significant differences between groups over time were further compared at each specific time point (12, 24, and 48 h). The association between strain parameters and mortality was evaluated with a univariate logistic regression model. Inter- and intra-observer variability was tested with Bland-Altman analyses. Data was analyzed using GraphPad Prism 6 (GraphPad Software, Inc. La Jolla, CA) as well as STATA 13 (StataCorp LP, College Station, TX). *P* value <0.05 was considered statistically significant.

## Results

### Hemodynamic parameters

**Fig. 2:**
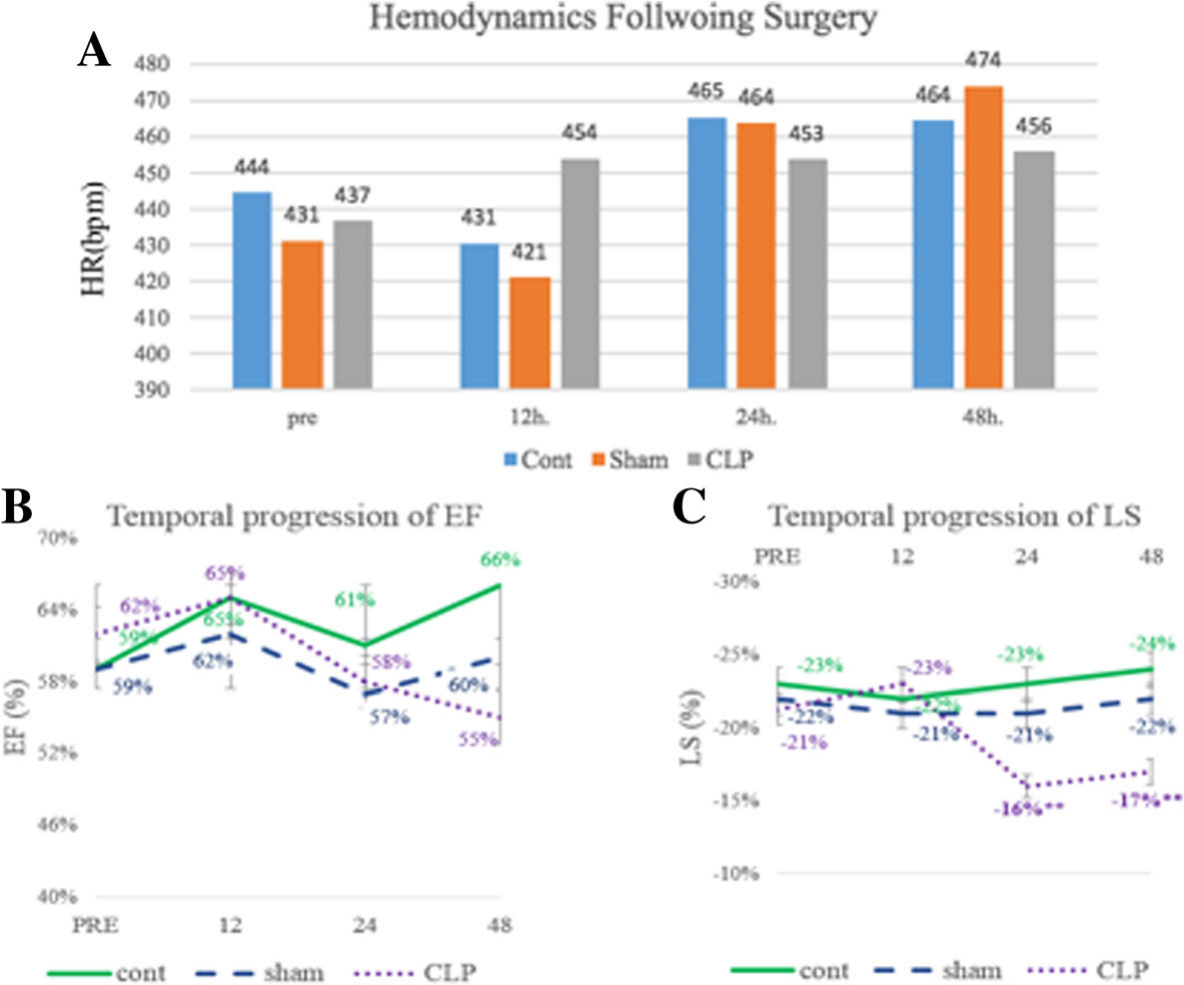
The temporal progression of heart rate, ejection fraction (EF), circumferential (CS), and longitudinal strain (LS) in C57BL/6J mice randomized to sham, control, and cecal ligation and puncture (CLP) groups. **a** Demonstrates an overall increase in heart rate overtime with no significant difference between the three groups. **b** and **c** Demonstrate notable depression in longitudinal strain (LS) in the CLP group at 24 and 48 h without change in the EF

Baseline weight and hemodynamic data collected was normally distributed (Table 1). After randomization, mice in the CLP group had an average weight of 22.4 ± 2.8 g and a heart rate of 436 ± 65 bpm [16] with no significant baseline differences across the three groups (CLP, sham, and control) (Table 1). Following CLP, a significant increase in HR was seen over time (*p* = 0.036) (Fig. 2a), but comparing the CLP group to sham and control yielded no significant difference at 24 h (*p* = 0.77) and 48 h (*p* = 0.74) following CLP (Table 2).

**Table 1:** Baseline characteristics and echocardiographic metrics for C57BL/6J mice

Variable	CLP (33)	Sham (25)	Control (10)	*p*
Weight (g)	22.42 ± 2.7	21.66 ± 3.31	20.5 ± 2.1	0.44
HR (bpm)	436 ± 65	431 ± 73	444 ± 62	0.87
2D Echo				
EF (%)	0.59 ± 0.1	0.59 ± 0.09	0.62 ± 0.09	0.57
Doppler				
E/A	1.6 ± 0.23	1.57 ± 0.18	1.57 ± 0.23	0.71
E/e’	25.2 ± 7.3	24.8 ± 5.0	27.3 ± 7.9	0.56
Strain				
CS (%)	−28.04 ± 5.74	−27.44 ± 5.7	−26.9 ± 4.4	0.87
CSR (%)	−11.45 ± 3.8	−10.5 ± 2.4	−9.6 ± 2.2	0.54
LS (%)	−21.3 ± 4.31	−22.23 ± 4.31	−23.43 ± 6.02	0.67
LSR (%)	−7.45 ± 1.9	−8.97 ± 2.96	−9.98 ± 4.7	0.04

*CLP* indicates cecal ligation and puncture, *CS* global circumferential strain, *CSR* peak systolic circumferential strain rate, *E/A* ratio of peak flow velocity across the mitral annulus during early and late diastole, *2D Echo* conventional echocardiography, *E/e’* ratio between mitral inflow and mitral annular excursion velocity during early diastole, *EF* ejection fraction, *LS* global longitudinal strain, *LSR* peak systolic longitudinal strain rate, *Strain* strain echocardiography

**Table 2:** Echocardiographic metrics at 24 and 48 h post-CLP for C57BL/6J mice

Parameter	CLP	Sham	Control	*p*
24 h post-CLP				
HR (bpm)	453 ± 65	463 ± 58	465 ± 46	0.77
2D Echo				
EF (%)	0.58 ± 0.11	0.57 ± 0.07	0.61 ± 0.08	0.68
E/A	1.45 ± 0.44	1.49 ± 0.23	1.49 ± 0.22	0.88
E/e’	19.1 ± 8.44	20.83 ± 6.65	22.9 ± 4.73	0.34
Strain				
CS (%)	−22.3 ± 5.7	−23.4 ± 10.75	−25.14 ± 3.18	0.26
CSR (%)	−9.6 ± 3.48	−12.14 ± 6.21	−10.69 ± 3.27	0.54
LS (%)	−16.6 ± 4.12	−21.01 ± 5.4	−22.5 ± 3.9	<0.01**
LSR (%)	−7.08 ± 2.52	−7.84 ± 2.23	−9.01 ± 2.36	0.08
48 h post-CLP				
HR (bpm)	455 ± 79	474 ± 39	464 ± 38	0.74
2D Echo				
EF (%)	0.55 ± 0.12	0.6 ± 0.09	0.66 ± 0.05	0.08
E/A	1.5 ± 0.12	1.49 ± 0.11	1.63 ± 0.32	0.73
E/e’	23.6 ± 8.04	20.7 ± 6.01	22.27 ± 7.15	0.7
Strain				
CS (%)	−24.4 ± 6.9	−26.3 ± 5.2	−28.3 ± 6.9	0.44
CSR (%)	−10.75 ± 3.38	−11.3 ± 3.29	−12.44 ± 2.56	0.55
LS (%)	−17.25 ± 4.72	−22.2 ± 5.8	−24.62 ± 7.12	0.04**
LSR (%)	−6.8 ± 2.31	−9.52 ± 2.31	−9.54 ± 2.41	0.036**

*CLP* indicates cecal ligation and puncture, *CS* global circumferential strain, *CSR* peak systolic circumferential strain rate, *E/A*, ratio of peak flow velocity across the mitral annulus during early and late diastole, *2D Echo* conventional echocardiography, *E/e’* ratio between mitral inflow and mitral annular excursion velocity during early diastole, *EF* ejection fraction, *LS*, global longitudinal strain, *LSR* peak systolic longitudinal strain rate *Strain* strain echocardiography. **CLP group is significantly different versus sham and control groups

### Conventional echocardiography parameters

All C57BL/6J wild-type mice in the study exhibited normal cardiac morphology at baseline. Mice in the CLP group demonstrated normal baseline indices of cardiac function (EF = 59 ± 10%) with no significant difference in EF across the three groups (Table 1) [17]. Following the surgery, EF in the CLP group was not significantly depressed when compared with baseline (baseline = 59 ± 10%, 12 h = 65 ± 2.4%, 24 h = 58 ± 11%, 48 h = 55 ± 12%; *p* = 0.58) (Fig. 2b). Furthermore, there was no significant change in EF across the three groups (CLP, sham, and control) (Table 2).

Tissue Doppler measures (e’ and E/e’) were normally distributed, whereas pulsed-wave Doppler metrics (E, A, and E/A) were negatively skewed. Following surgery, there was no significant difference in diastolic function (E/A and E/e’) across the three groups (CLP, sham, and control) at 24 and 48 h (Table 2).

### Strain echocardiography

In the C57BL/6J wild-type mice, baseline strain measurements (CS and LS) were normally distributed whereas strain rate measurements (CSR and LSR) were negatively skewed. When evaluating temporal progression of cardiac function using SE, significant depression in LS (pre = −21.3 ± 4.31% vs. 24 h = −16.6 ± 4.12%; *p* < 0.01) and CS (pre = −28.04 ± 5.74% vs. 24 h = −22.3 ± 5.7%; *p* < 0.01), was detected in the CLP group when compared to baseline (Fig. 2b). Further evaluation of SE metrics across the three groups at 24 h revealed significant LS depression in the CLP mice (−16.6 ± 4.12%) when compared to sham (−21.01 ± 5.4%) and control (−22.5 ± 3.9%; *p* = 0.01) (Table 2). At 48 h following CLP, significant depression in both longitudinal strain and strain rate (LS and LSR) was identified in the CLP mice (LS = −17.25 ± 4.72%, LSR = −6.8 ± 2.31) when compared to sham (LS = −22.2 ± 5.8%, LSR = −9.52 ± 2.31) and control (LS = −24.62 ± 7.12%, LSR = −9.54 ± 2.41) (*p* = 0.04, *p* = 0.036) (Table 2).

Furthermore, univariate logistic regression analysis revealed a significant association between increased odds of mortality and reduced LS at 24 h (OR = 1.23, *p* = 0.04) and reduced CS at 24 h (OR = 1.14, *p* = 0.05). No significant correlation was found between EF and mortality in this model (Additional file 2: Table S3).

### Cytokine profile

**Fig. 3:**
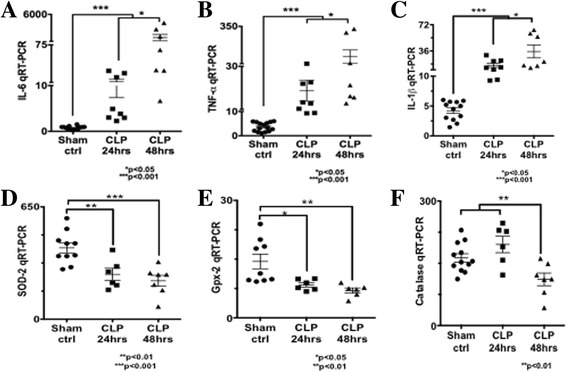
Quantitative real-time PCR of pro-inflammatory cytokines (TNF-α, IL-1, and Il-6) and mitochondrial free radical scavengers (SOD-2, GPX-2, Catalase) at 24 and 48 h following CLP. Significant increase in TNF-α, IL-1, and Il-6 is seen at 24 and 48 h in the CLP group when compared to sham and control (**a**–**c**). Significant decrease in mitochondrial ROS scavengers is seen at 24 and 48 h post-surgery in the CLP group when compared to sham and control (**d**–**f**)

Cytokine profile from the cardiac tissue of C57BL/6J mice demonstrated a significant increase in pro-inflammatory cytokines (TNF-α, IL-1β, and IL-6) in the CLP group when compared to sham and control (Fig. 3a–c). This upregulation in inflammatory cytokine expression temporally coincides with the cardiac dysfunction seen by SE in the mice subjected to CLP.

### Mitochondrial free radical scavenger expression and cardiomyocyte oxidative stress

**Fig. 4:**
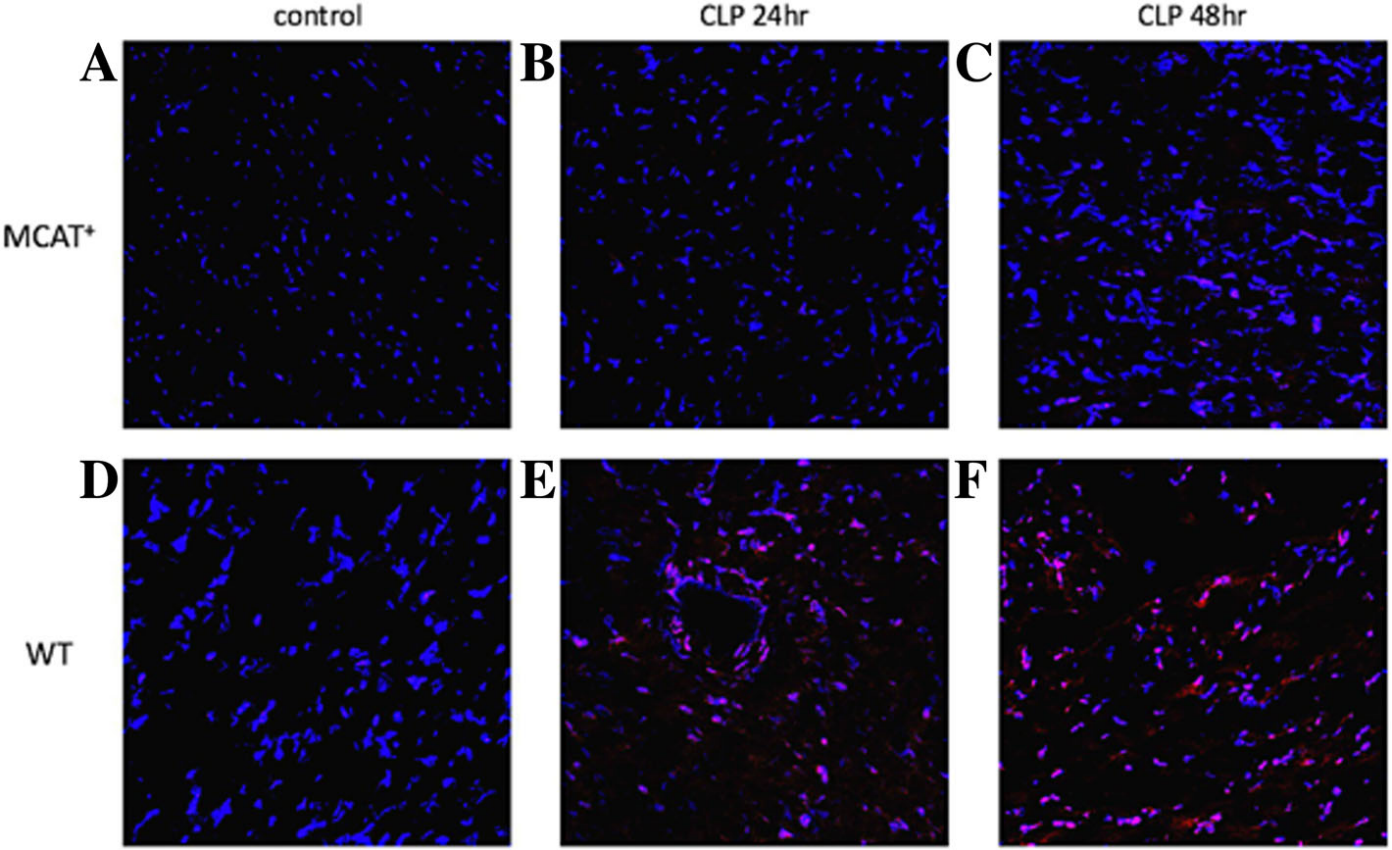
Photomicrographs of heart sections stained with dihydroethidium (DHE) in *red* and DAPI in *blue* (merged images) from septic MCAT ^+/+^ (**a**, **b**, **c**) and WT mice (**d**, **e**, **f**). Significant increase in level of oxidative stress (signified by increased uptake of DHE) in the WT mice at 24 (**e**) and 48 h (**f**) following CLP. Minimal signs of oxidative stress are seen the MCAT ^+/+^ mice following CLP

The cardiac dysfunction identified by SE in the septic mice also coincides with a significant decrease in cardiomyocyte expression of redox scavengers (SOD-2, Gpx-2, and catalase) at 24, and 48 h following CLP (Fig. 3d–f). These findings correlate with a significant increase in tissue DHE uptake determined by confocal microscopy at 24 and 48 h post-CLP (Fig. 4d–f) suggesting an increase in cardiomyocyte oxidative stress in the mice subjected to CLP.

### Mitochondrial targeted antioxidant upregulation using MCAT^+/+^ transgenic mice

To evaluate the influence of ROS scavenger upregulation on oxidative stress as well as cardiac function in sepsis, SE and oxidative stress were evaluated between a group of C57BL/6J MCAT^*+/+*^ transgenic mice (*n* = 12), which overexpress mitochondrial-targeted catalase and C57BL/6J WT mice (*n* = 14) following CLP. Both groups underwent conventional and SE pre-, 24, and 48 h post-surgery (Table 3).

**Table 3:** Association between 24 h echocardiographic metrics and mortality for C57BL/6J mice

Parameter	OR	*p*	CI
Heart rate	0.99	0.85	0.98–1.02
Ejection fraction	1.06	0.179	0.02–1.8
E/A	0.4	0.35	0.06–2.72
E/e’	1.07	0.17	0.97–1.20
LS	1.23	0.04*	1.01–1.51
CS	1.14	0.05*	1.02–1.32
LSR	1.49	0.108	0.92–2.42
CSR	1.12	0.285	0.91–1.38

*CI* indicates confidence interval, *CS*, global circumferential strain, *CSR* peak systolic circumferential strain rate, *E/A* ratio of peak flow velocity across the mitral annulus during early and late diastole, *E/e’* ratio between mitral inflow and mitral annular excursion velocity during early diastole, *LS* global longitudinal strain, *LSR* peak systolic longitudinal strain rate, *OR* odds ratio. *Significant association with mortality based on univariate logistic regression analysis

When evaluating cardiac function via SE between the two groups over time, significant difference in both LS (*p* = 0.01) as well as CS (*p* > 0.01) was identified. In particular, C57BL/6J WT mice demonstrated a significant decrease in longitudinal strain (LS) at 24 h (−23 ± 5% vs. −15 ± 4% *p* < 0.01) and 48 h (−23 ± 7% vs. −16.5 ± 3.9% *p* = 0.04) post-CLP while C57Bl/6 *MCAT* ^*+/+*^ transgenic mice had preserved cardiac function by SE (Table 4). Furthermore, C57BL/6J WT mice demonstrated a dramatic increase in level of global oxidative stress, evidenced by the increased uptake of DHE staining at 24 and 48 h following CLP while C57Bl/6 *MCAT* ^*+/+*^ transgenic mice demonstrated a minimal increase in oxidative stress (Fig. 4a–c).

**Table 4:** MCAT and Wildtype Mice Echocardiographic Metrics at 24 and 48 h post-CLP

	CLP (WT)	CLP (MCAT)	Sham (WT)	Sham (MCAT)	*p*
24 h post-CLP					
HR (bpm)	451 ± 69	405 ± 71	426 ± 78	454 ± 79	0.64
2D Echo					
EF (%)	58.3 ± 15.5	61 ± 10.6	61 ± 8	52.1 ± 8	0.17
E/A	1.34 ± 0.61	1.43 ± 0.18	1.49 ± 0.22	1.37 ± 0.19	0.85
E/e’	16.19 ± 6.9	27.9 ± 6.95	22.9 ± 4.73	20.1 ± 4.06	0.01
Strain					
CS (%)	−20.9 ± 7.3	−26.4 ± 8.8	−25.14 ± 3.18	−27.5 ± 4.7	0.1
CSR (%)	−10.32 ± 4.4	−11.67 ± 7.19	−10.69 ± 3.27	−10.2 ± 1.52	0.76
LS (%)	−15.4 ± 4.1	−21.5 ± 4.9	−22.5 ± 3.9	−22.2 ± 2.8	0.01
LSR (%)	−6.9 ± 3.75	−8.9 ± 1.8	−9.01 ± 2.36	−10.33 ± 3.74	0.01
48 h post-CLP					
HR (bpm)	420 ± 74	408 ± 58	466 ± 73	440 ± 34	0.71
2D Echo					
EF (%)	55.2 ± 1.06	66 ± 9.7	0.61 ± 0.08	61.6 ± 6.98	0.14
E/A	1.49 ± 0.14	1.44 ± 0.08	1.49 ± 0.22	1.42 ± 0.15	0.65
E/e’	32.41 ± 7.25	23.41 ± 7.25	22.9 ± 4.73	27.15 ± 7.44	0.22
Strain					
CS (%)	−20.6 ± 7.5	−29.4 ± 4.7	−25.14 ± 3.18	−31.7 ± 3.5	0.01
CSR (%)	−8.87 ± 3.02	−13.6 ± 3.07	−10.69 ± 3.27	−12.1 ± 1.58	0.02
LS (%)	−16.5 ± 3.9	−21.6 ± 4.8	−22.5 ± 3.9	−24.15 ± 7.7	0.01
LSR (%)	−9.06 ± 3.86	−10.79 ± 2.71	−9.01 ± 2.36	−6.69 ± 1.73	0.5

*CLP* indicates cecal ligation and puncture, *CS* global circumferential strain, *CSR* peak systolic circumferential strain rate, *E/A* ratio of peak flow velocity across the mitral annulus during early and late diastole, *2D Echo* conventional echocardiography, *E/e’* ratio between mitral inflow and mitral annular excursion velocity during early diastole, *EF* ejection fraction, *LS* global longitudinal strain, *LSR* peak systolic longitudinal strain rate, *Strain* strain echocardiography

## Discussion

This is the first study to illustrate an association between SE and increased myocardial oxidative stress as well as mortality in a well-established sepsis animal model. Furthermore, the use of targeted ROS scavengers to abrogate myocardial dysfunction emphasizes the role of myocardial oxidative stress in the development of cardiomyopathy in sepsis. Lastly, SE detected significant depression in myocardial contractility, despite a normal EF supporting the recent clinical literature which suggests improved sensitivity of SE for subtle cardiac dysfunction in sepsis [18].

The current literature on the utility of SE focuses on ischemic and hypertrophic cardiomyopathy. Studies by Park and colleagues [19] have demonstrated an association between regional SE depression and infarct size in patients with ischemic cardiomyopathy. Furthermore, SE has been used to predict left ventricular remodeling [19] and response to reperfusion strategies [20].

However, the number of studies that have evaluated septic shock using SE are limited. In a recent study by Orde and colleagues, SE revealed significant bi-ventricular dysfunction in sepsis that is associated with Sequential Organ Failure Assessment score and 3- and 6-month mortality [21]. Dalla and colleagues demonstrated a significant decrease in strain measurement (LS) in septic patients when compared to a non-septic trauma cohort, suggesting that cardiac dysfunction identified by SE in sepsis is independent of the cardiomyopathy that can be seen from the hyperadrenergic state found in critical illness [22]. While the findings of this investigation are consistent with the current literature, this is the first study to demonstrate an association between SE depression, cardiac oxidative stress and mortality in a well-established animal model.

The myopathy identified by SE in this sepsis animal model was found independent of the variability in afterload. Although SE is assumed to be less load dependent than conventional metrics of cardiac function, it is conceiveable that decreased systemic vascular resistance in the septic group can falsely elevate strain and EF. Therefore, the cardiac dysfunction identified by SE in the CLP group might be underestimated in the setting of low systemic vascular resistance.

### Strain depression in sepsis is associated with upregulation of pro-inflammatory cytokines

While the presence and significance of SIMD is well established, the mechanistic pathways behind this disease remain unclear. Currently, a variety of circulating inflammatory mediators (TNF-α, IL-1β, and IL-6) have been implicated as possible myocardial depressant factors (MDF) [23]. This study demonstrates an association between significant cardiac depression by SE and increase in expression of pro-inflammatory cytokines (TNF-α, IL-6, and IL-1β) further supporting the current literature regarding the role of pro-inflammatory cytokines in the development of myopathy in sepsis.

### Strain depression in sepsis is associated with increased cardiomyocyte oxidative stress

Previous studies have demonstrated the impact of sepsis-induced mitochondrial oxidative stress on mitochondrial DNA damage [24], dysfunction of the electron transport chain [25] and reduction of ATP synthesis [26]. While these findings have been displayed using a variety of validated assays including DHE staining [24–28], non-invasive metrics that detect early ROS mediated contractile dysfunction have been lacking. This study identified a temporal association between cardiac dysfunction detected by SE and cardiomyocyte oxidative stress. These findings further support the impact of ROS on the development of myopathy in sepsis and display the possible utility of SE in the identification of subtle, early contractile dysfunction in sepsis.

### Influence of ROS scavengers on oxidative stress and cardiac mechanics

While sepsis induced mitochondrial oxidative stress has been recognized as a significant mechanistic pathway in SIMD, the role of mitochondrial targeted ROS scavengers in abrogating mitochondrial oxidative stress has not been adequately explored. Previous studies using global antioxidants as potential therapeutic targets in sepsis have fallen short, likely due to compartmentalized cellular effects of ROS and limited antioxidant penetration into the mitochondria [29]. Although augmentation of mitochondrial-oriented free radical scavengers such as MCAT and SOD-2 have shown promising results in animal models of ischemic cardiomyopathy as well as hypertensive heart failure [30, 31], they have not been adequately explored in the context of sepsis.

This study demonstrates preservation of cardiac function as well as limited oxidative stress in *MCAT* ^*+/+*^ transgenic mice subjected to sepsis. *MCAT* ^*+/+*^ transgenic mice have a CMV enhancer beta-actin promoter driving the expression of a human catalase (*CAT*) gene with a mitochondrial localization sequence. *MCAT* ^*+/+*^ mice are thought to have increased catalase activity (up to 50 times higher) in heart and skeletal muscle when compared to wild type mice [32]. Results from this study coincides with the current literature which demonstrates the cardioprotective benefit of MCAT upregulation in animal models heart failure [30, 31] and suggests the possibility of designing effective therapeutic strategies to modulate cardiac oxidative stress in sepsis (Table 4).

### Strain and outcomes

Previous studies have explored the link between SE depression and clinical outcomes in adults with sepsis [20]. Using SE, Orde and colleagues [20] demonstrated increased odds of mortality (OR = 1.1, *p* = 0.02) in patients with sepsis who developed right ventricular strain depression. Chang and colleagues [33] also demonstrated that a threshold of left ventricular LS ≥ −13% is a good predictor of mortality (ROC = 0.79, hazard ratio = 4.21, *p* = 0.01) in a cohort of septic adults. The findings in this study agree with available clinical literature which suggests an association between SE depression and poor outcomes in sepsis.

### Limitations

Some limitations in these findings need to be acknowledged. In particular, it is well known that some degree of variability is inherent to the CLP animal model [34]. Although CLP is a widely accepted animal model of sepsis, the severity of the disease is dependent upon multiple factors [34]. This study utilized a consistent CLP protocol with regard to the length of the cecal ligation, number, and size of the puncture. Furthermore, to decrease the chance of variability, one investigator (BH) performed all the surgical procedures.

Another limitation of this study is the technical variability behind attaining consistent apical views for tissue and pulse wave Doppler imaging in a mouse. Attaining consistent apical views in mice can be extremely difficult since it can be influenced by the orientation of an animal’s heart as well as that of the transducer. While the apical view is essential for the tissue Doppler imaging assessment of longitudinal strain and strain rate, the advent of more sophisticated speckle-tracking strain post-processing software has been able to overcome this limitation. Unlike tissue Doppler evaluation of strain and strain rate which evaluates mitral annular motion along the long axis of the heart only, speckle-tracking strain can track myocardial tissue motion in the radial direction. This has made the parasternal long axis view a viable option for evaluating longitudinal speckle-tracking strain and strain rate. The speckle-tracking strain software used in this study (VevoStrain 2D FUJIFILM, VisualSonics) has been previously utilized to assess longitudinal strain from parasternal long axis view in several cardiac injury models including ischemic and hypertrophic cardiomyopathy [35–37]. Furthermore, to limit interobserver variability, the echocardiography was performed by only one researcher utilizing a standard protocol for image acquisition and quality control.

Lastly, the use of inhaled isoflurane as a sedative during the surgical procedure and echocardiography could potentially confound our measurements of contractile function. Inhaled isoflurane at high levels can reduce left ventricular systolic and diastolic function and can induce vasoplegia [38]. In this study, the dose and the length of isoflurane administration was similar in all three experimental groups (CLP, sham, and control) thus limiting confounding by anesthesia.

## Conclusions

In this study, cardiac dysfunction demonstrated by using SE was associated with repression of mitochondrial ROS scavengers and increased cardiomyocyte oxidative stress. Strikingly, SE identified cardiac dysfunction despite no difference in EF between experimental groups. This study also demonstrated a strong correlation between cardiac depression, as detected by SE (LS, % and CS, %) and mortality. These findings suggest that SE can be used as a sensitive diagnostic tool in the evaluation of myocardial dysfunction in sepsis. Furthermore, modulation of mitochondrial oxidative stress could play a beneficial role in the prevention of this devastating complication of sepsis.

## Additional files

Additional file 1: Table S1. Primer table for inflammatory cytokines as well as ROS scavengers. (DOCX 12 kb)

Additional file 2: Table S3. MCAT and Wildtype Mice Baseline Echocardiographic Metrics. (DOCX 14 kb)

## Abbreviations

CLP: Cecal ligation and puncture
CS: Global circumferential strain
CSR: Peak systolic circumferential strain rate
EF: Ejection fraction
FS: Fractional shortening
LS: Global longitudinal strain
LSR: Peak systolic longitudinal strain rate
SE: Strain echocardiography
SIMD: Sepsis-induced myocardial dysfunction
