# Supplementary material for: Myocardial oxidative stress correlates with left ventricular dysfunction on strain echocardiography in a rodent model of sepsis
Source: Intensive Care Med Exp. 2017 Apr 12;5:21. doi: 10.1186/s40635-017-0134-5 (PMC5389950; doi:10.1186/s40635-017-0134-5)
Supplement: Supplementary file 1 — Primer table for inflammatory cytokines as well as ROS scavengers. (DOCX 12 kb) [file 40635_2017_134_MOESM1_ESM.docx]

Table S1: Primer table for inflammatory Cytokines as well as ROS scavengers

| **Gene** | **Forward sequence** | **Reverse sequence** | **Amplicon**  **Size (bP)** |
| --- | --- | --- | --- |
| Catalase | GTGCCCCCAACTATTACCCC | TCACACAGGCGTTTCCTCTC | 184 |
| Gpx-2 | TCAATGGGCAGAACGAGCAT | CGCACGGGACTCCATATGAT | 118 |
| IL-6 | GGCTAAGGACCAAGACCATCCAA | TCTGACCACAGTGAGGAATGTCCA | 138 |
| IL1β | AGTGTGGATCCCAAGCAATACCCA | TGTCCTGACCACTGTTGTTTCCCA | 175 |
| SOD-2 | GTAGGGCCTGTCCGATGATG | CGCTACTGAGAAAGGTGCCA | 130 |
| Rplp0 | GGCGACCTGGAAGTCCAACT | CCATCAGCACCACAGCCTTC | 143 |
| TNF-α | CATCTTCTCAAAATTCGAGTGACAA | TGGGAGTAGACAAGGTACAACCC | 175 |

Primer sequence utilized for qPCR of inflammatory cytokines as well as redox scavengers. GPX-2 indicates Intracellular Glutathione-peroxidase, SOD-2 indicates Mitochondrial Superoxide dismutase
