# Supplementary material for: Myocardial oxidative stress correlates with left ventricular dysfunction on strain echocardiography in a rodent model of sepsis
Source: Intensive Care Med Exp. 2017 Apr 12;5:21. doi: 10.1186/s40635-017-0134-5 (PMC5389950; doi:10.1186/s40635-017-0134-5)
Supplement: Supplementary file 2 — MCAT and Wildtype Mice Baseline Echocardiographic Metrics. (DOCX 14 kb) [file 40635_2017_134_MOESM2_ESM.docx]

Table S3. MCAT and Wildtype Mice Baseline Echocardiographic Metrics

|  | CLP  (WT) | CLP  (MCAT) | sham  (WT) | sham  (MCAT) | P |
| --- | --- | --- | --- | --- | --- |
| HR (bpm) | 424 ± 58 | 417 ± 67 | 398± 75 | 448 ± 71 | 0.64 |
| 2D Echo: |  |  |  |  |  |
| EF (%) | 60 ± 9.6 | 57± 9.2 | 68 ± 5.2 | 62.3± 10.5 | 0.61 |
| E/A | 1.61 ± 0.22 | 1.42± 0.15 | 1.49 ± 0.22 | 1.3 ± .14 | 0.06 |
| E/e' | 24.01 ± 5.6 | 26.7 ± 5.7 | 22.9 ± 4.73 | 25.6± 9.4 | 0.51 |
| Strain |  |  |  |  |  |
| CS (%) | -25.2± 4.5 | -25.9 ± 5.3 | -25.14 ± 3.18 | -27.9 ± 3.6 | 0.53 |
| CSR (%) | -9.93 ± 2.69 | -9.31 ± 2.25 | -10.69 ± 3.27 | -11.22 ± 3.59 | 0.08 |
| LS (%) | -20.2 ± 2.4 | -21.8 ± 3.3 | -22.5 ± 3.9 | - 20.2 ± 2.4 | 0.11 |
| LSR (%) | -6.82 ± 1.72 | -8.62 ± 1.8 | -9.01 ± 2.36 | -9.84 ± 2.33 | 0.03 |

CLP indicates cecal ligation and puncture; CS, global circumferential strain; CSR, peak systolic circumferential strain rate; E/A, ratio of peak flow velocity across the mitral annulus during early and late diastole; Echo 2D, conventional echocardiography; E/e', ratio between mitral inflow and mitral annular excursion velocity during early diastole; EF, ejection fraction; LS, global longitudinal strain; LSR, peak systolic longitudinal strain rate; Strain, strain echocardiography.
